# Social network analysis of stakeholders in China's hierarchical medical system: toward a collaborative governance framework for enhanced integration

**DOI:** 10.3389/fpubh.2026.1720264

**Published:** 2026-03-11

**Authors:** Qiumao Cai, Xiatong Ke, Yuyao Li, Huatang Zeng, Fang Du, Liqun Wu, Jun Xu

**Affiliations:** 1 School of Public Health, Southern Medical University, Guangzhou, Guangdong, China; 2 Nanfang Hospital, Southern Medical University, Guangzhou, Guangdong, China; 3 Department of Assessment, Shenzhen Health Development Research and Data Management Center, Shenzhen, Guangdong, China; 4 Vanke School of Public Health, Tsinghua University, Beijing, China; 5 Southern Medical University Hospital of Integrated Traditional Chinese and Western Medicine, Southern Medical University, Guangzhou, Guangdong, China

**Keywords:** China, governance framework, Hierarchical Medical System (HMS), Social Network Analysis (SNA), stakeholders collaboration

## Abstract

**Background:**

The Hierarchical Medical System (HMS) in China aims to streamline healthcare delivery through structured patient referrals across primary, secondary, and tertiary care. However, the weak collaboration among stakeholders, such as government agencies, medical institutions, and patients has become a significant barrier to its effective implementation.

**Methods:**

This study employed Social Network Analysis (SNA) to explore the social network characteristics and functional positioning of key stakeholders in the HMS. A systematic literature review was conducted to identify 14 primary stakeholders, followed by a survey of 631 experts to assess the relationships between these stakeholders. The expert judgments were used to construct a relationship matrix, which served as the basis for network analysis.

**Results:**

Our findings revealed that stakeholder collaboration within the HMS operates as a moderately connected network, characterized by limited integration and low participation from both patients and primary healthcare institutions. This lack of engagement is a primary barrier to the effective implementation of the system. Among the core stakeholders, including the Health Committee, Finance Bureau, Development and Reform Commission, and Healthcare Security Administration, there was insufficient cohesion and coordination, which weakened the overall network. This lack of integration significantly hampers policy synergy and reduces implementation efficiency.

**Conclusions:**

A collaborative governance framework is proposed to address these challenges. By fostering greater engagement among stakeholders and empowering patients to voice their needs, the system can achieve better integration, equity, and responsiveness. This framework aims to improve system efficiency and policy implementation, facilitating more effective governance and better healthcare outcomes.

## 1 Introduction

China's healthcare system faces a striking paradox: while primary medical institutions receive relatively few patients, tertiary hospitals are chronically overcrowded. Addressing this “wartime state” in tertiary hospitals and establishing a more rational care delivery structure have become pressing priorities for healthcare reform (1, 2). A major contributing factor is patients' lack of trust in primary care, leading many to bypass it and seek care directly at tertiary hospitals (3). This behavior exacerbates the imbalance in medical resource allocation.

To address these challenges, the Chinese government issued the Guiding Opinions of the General Office of the State Council on Pushing Forward the Building of the Hierarchical Medical System (HMS) in September 2015 (4). HMS is designed to foster alliances between primary medical institutions and tertiary hospitals within the same region, enabling the downward transfer of high-quality medical resources, enhancing primary care service capacity, encouraging voluntary first-contact care at the primary level, and reducing unnecessary patient flows to tertiary hospitals (5). A further goal is to streamline referral procedures, particularly for chronic and convalescent patients, thereby establishing an orderly bidirectional referral system across healthcare tiers (6).

In recent years, multiple HMS reform models have been piloted across China, including the Luohu Model in Shenzhen (7); Shanghai's “1+1+1” healthcare organization model (8); the Anhui Tianchang Medical Community Model (9); and the Xiamen co-management of chronic disease under three disciplines model, which was developed by the Xiamen Chronic Disease Center (Xiamen, China) (10), as well as the cross-regional specialty alliance model, among others. However, the implementation of HMS has not yielded the expected results, and its specific path to success remains unclear (11). Several key issues persist:

(1) **Patient-centered care:** current HMS approaches mainly use administrative “grading” to triage patients, rather than focusing on creating a “patient-centered” system, leading to a lack of stable relationships between patients and doctors or between different levels of medical institutions (12).
(2) **Referral system issues:** there is no clear referral standard between medical institutions of different levels in China; tertiary hospitals within medical associations have a siphoning effect on the sources of disease and medical care at the grassroots level, and the downwards transfer of patients is not smooth (13).
(3) **Family doctor services:** many contracted family doctor services remain formalities. Primary care physicians often struggle to decide whether referrals are necessary, further impeding the smooth upward referral of patients (14).
(4) **Weak medical resources at the primary level:** while the intent is to redistribute resources, the decrease in high-quality medical resources at the grassroots level has not been as pronounced as expected. As a result, primary healthcare institutions continue to lack the diagnostic and treatment capacity to earn patient trust, preventing them from serving as the first point of contact (15).
(5) **Medical insurance reimbursement:** unlike mandatory gatekeeper systems in countries like Britain and Germany, China's HMS restricts patient bypassing of primary care through differential reimbursement ratios in medical insurance. However, the current system is insufficient to guide patient decisions, and it fails to maximize the incentive role of medical insurance (16).

These issues involve multiple stakeholders, including health committees, healthcare security administrations, medical institutions at various levels, and patients (17). Effective implementation of HMS hinges on these stakeholders working together to facilitate two-way referrals and ensure continuous, coordinated, whole-life-cycle healthcare services. However, stakeholders often have differing motives, interests, and readiness to implement policies, resulting in conflicts at both the cognitive and operational levels.

Given this complexity, there is an urgent need to establish an organic system of collaborative governance among stakeholders. This would enable a more balanced allocation of responsibilities and interests, leading to an effective and rational governance structure.

The relationships among stakeholders in HMS form a complex network with social network characteristics (18). Social network analysis (SNA) can help identify key stakeholders, analyze their relationships and cooperation modes, and quantify the strength, efficiency, and coordination of these interactions (19). This method can significantly enhance the coordination of stakeholders and improve the successful implementation of HMS (20). The ultimate goal is to provide actionable insights for government agencies and healthcare departments to guide the multi-level, multi-collaborative governance of the healthcare system.

## 2 Methods

**Figure 1:**
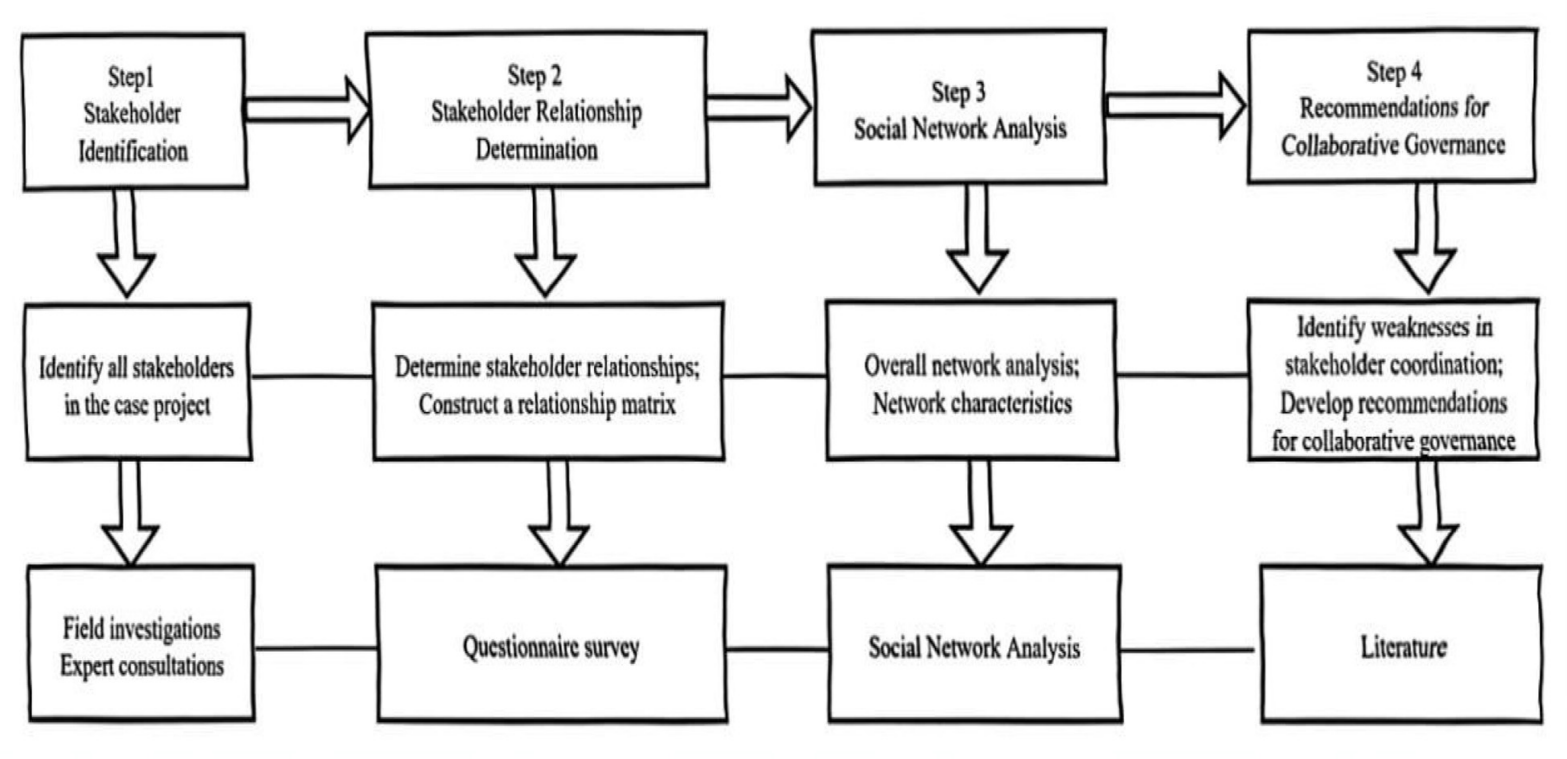
Research process.

As shown in Figure 1, the process began with the identification and classification of stakeholders involved in the case project, achieved through field investigations and expert consultations. Next, a questionnaire survey was conducted to assess the intensity of relationships among stakeholders, leading to the development of a relationship matrix. Subsequently, social network analysis (SNA) was employed to examine the network at two hierarchical levels. To ensure analytical rigor, metrics were categorized into: (a) **Network-level indicators:** including network density, network size, network connectivity, and centralization indices, used to characterize the overall structural properties and integration of the HMS. (b) **Node-level indicators:** including standardized degree centrality, betweenness centrality, and closeness centrality. These normalized indices allow for a comparative assessment of the relative influence and communication advantage of individual stakeholders regardless of network size. Detailed definitions for each indicator are provided in Table 1.

**Table 1:** Indicators for social network analysis.

**Level**	**Indicator**	**Definition**	**Purpose**
Network-level	Network density	The ratio of actual connections to all possible connections in the network.	Measures the overall closeness, cohesion, and interaction strength among stakeholders.
	Core–periphery structure	A network pattern distinguishing a tightly connected “core” group from a loosely connected “peripheral” group.	Reveals the concentration of power and distinguishes central coordinators from marginal executors.
	Network centralization	A measure of the extent to which the entire network is organized around a few central nodes.	Reflects the hierarchical nature of the system and its structural dependence on specific institutions.
Node-level	Normalized degree centrality	The number of direct links a node has, standardized by the maximum possible connections (N−1).	Identifies “power centers” with direct access to resources; normalization allows for cross-network comparison.
	Normalized betweenness centrality	The frequency with which a node lies on the shortest path between other pairs of nodes, standardized.	Identifies stakeholders acting as **“Brokers”** or bridges, revealing their control over information and resource flow.
	Normalized closeness centrality	The inverse of the sum of distances from a node to all others, standardized.	Measures the efficiency of an institution in reaching others and its independence from the control of intermediaries.

This study utilized UCINET 6.0 to conduct a social network analysis of stakeholders within the HMS, and employed PowerPoint software to visualize the stakeholder relationship network. To enhance collaborative governance among multiple actors, it is essential to systematically examine stakeholder relationships from the perspectives of overall structure, subgroup characteristics, and individual stakeholder positions. To evaluate the overall features of the stakeholder network, indicators such as network density and average path length were used to assess the closeness of connections and communication efficiency (21). To further explore the interactions among stakeholders, core-periphery analysis and clique analysis were conducted to identify the core stakeholder groups at various stages of HMS development and to reveal which stakeholders maintain closer ties with each other (22). In addition, three commonly used centrality measures, namely degree centrality, betweenness centrality, and closeness centrality, were applied to determine the influence and roles of stakeholders within the network (23). Centrality analysis offers valuable insights into which actors hold control over resources, serve as coordinators, or enjoy advantages in information flow throughout the HMS governance process.

## 3 Construction of stakeholder network in HMS

The stakeholder network in the HMS comprises nodes, representing stakeholders, and edges, indicating the strength or presence of relationships between these stakeholders. To construct such a network, it is crucial to first identify the relevant stakeholders, followed by an assessment of their interrelationships, which is captured in a stakeholder matrix. This matrix subsequently serves as the foundation for analyzing network.

### 3.1 Stakeholder identification

To systematically identify key stakeholders involved in China's HMS, we conducted a structured literature review. Four databases were searched: CNKI, ScienceDirect, Web of Science, and Pubmed. The search strategy combined Boolean operators as follows: (“stakeholder” OR “interest group” OR “actor”) AND (“health system reform” OR “healthcare governance” OR “health management”) AND (“China” OR “Chinese health system”).

The search was restricted to studies published between 2015 and 2025, corresponding to the period following the national rollout of HMS-related reforms. Only peer-reviewed journal articles written in English or Chinese were considered.

Inclusion criteria were: (1) explicit focus on China's healthcare system or HMS-related reforms; (2) analysis of governance structures, policy implementation, or inter-organizational relationships; and (3) explicit identification or discussion of stakeholder roles, interests, or interactions. Exclusion criteria were: (1) purely clinical or technical studies without governance relevance; (2) studies on non-Chinese health systems; (3) commentaries, editorials, or news reports lacking analytical content; and (4) duplicate publications.

The database search initially yielded a larger pool of records. After removal of duplicates, titles and abstracts were screened, followed by full-text assessment based on the above criteria. Finally, 13 articles met all eligibility requirements and were retained for stakeholder extraction. These studies consistently highlighted a core group of governmental authorities, healthcare institutions at different levels, and service users.

Based on these 13 articles, we summarized 14 stakeholder categories and their roles in the HMS, as presented in Table 2.

**Table 2:** HMS stakeholders identification.

**Stakeholder**	**Role in the HMS**	**Literature**
1. Government (central/local health authorities)	Macro-level policy design and overall leadership of HMS reform	(34, 35)
2. Finance bureau	Financial support for healthcare institutions and insurance fund	(5, 34)
3. Development and reform Commission (NDRC)	Oversees healthcare pricing and policy implementation	(36, 37)
4. Bureau of human resources and social security	Oversees healthcare personnel compensation, social protection, and recruitment	(36, 38)
5. Establishment (staffing) department	Allocates staffing resources to support HMS development	(36, 38)
6. Health committee	Guides referrals, ensures equitable resource allocation and service coordination	(39)
7. Medical products administration	Regulates drug policies, procurement, and access within different facility levels	(39, 40)
8. Healthcare security administration	Manages medical insurance, fund utilization, and regulatory oversight	(34)
9. Tertiary hospitals	Treat complex and severe cases referred from lower levels	(41)
10. Secondary hospitals	Diagnose and treat moderately complex diseases; assist with diagnostics and surgery	(41)
11. Community health centers	Manage residents' health, initial diagnosis, and common illness treatment	(41, 42)
12. Township health centers	Provide care for moderately complex illnesses and support basic procedural services	(40)
13. Village clinics	Offer basic healthcare and health monitoring in rural communities	(40)
14. Patients and patient-related personnel	End-users and primary beneficiaries whose behaviors and needs shape HMS outcomes	(43, 44)

### 3.2 Stakeholder judgement

To further validate and refine the stakeholder categories identified through the literature review, we conducted a structured expert survey (the first round). Experts in public health, healthcare policy, health system governance, and HMS reform practice were invited to assess the relevance of each stakeholder category derived from the literature. Rather than asking experts to generate stakeholders *de novo*, we asked them to evaluate the importance of each pre-identified category by responding to the question: “*Do you believe this stakeholder plays a significant role in achieving the goals of the HMS?”*.

For each stakeholder category, we calculated the proportion of experts who answered “yes.” Categories were retained in the HMS stakeholder network if more than 50% of respondents considered them relevant. This >50% rule was used as a pragmatic screening criterion to filter out marginal or highly context-specific actors, rather than as a definitive measure of stakeholder importance. Its limitations are acknowledged and further discussed in the Limitations section. In addition, experts were encouraged to propose any stakeholders they regarded as missing from the initial list, and these suggestions were reviewed and, where appropriate, integrated into the final set of stakeholder categories. This process ensured that the final stakeholder list was grounded in both the published literature and collective expert judgement, thereby reducing the risk of arbitrary selection based on a single source.

For the purposes of the network analysis, all identified stakeholder categories were modeled as nodes in a single one-mode network. This representation implies structural equivalence in a graph-theoretic sense, but does not assume substantive equivalence in terms of authority, resources or modes of interaction. In practice, government agencies, healthcare institutions and patients differ markedly in their decision-making power, organizational capacity and the ways in which they engage with the HMS. The network should therefore be interpreted as a map of perceived influence relationships among heterogeneous actor types, rather than as evidence that all nodes participate in the system in the same way.

### 3.3 Construction of the stakeholder adjacency matrix

To assess the relationships among stakeholders, a structured questionnaire was administered to the same group of experts (the second round). For each potential relationship between two stakeholders, experts were asked to answer the binary question: “*Do you believe this stakeholder plays a significant role in achieving the goals of the HMS?”* Experts responded with either “Yes” or “No” to indicate whether they believed the relationship between the stakeholders was significant.

This binary approach allowed us to construct an adjacency matrix, where relationships were coded as present (1) if more than 70% of respondents answered “Yes” for a given pair, and absent (0) if fewer than 70% of experts considered the relationship to be significant. The 70% threshold was used as a pragmatic consensus rule to ensure that only the most widely acknowledged relationships were included in the analysis. This threshold was not intended as an absolute measure of the importance of the relationships but as a way to capture the consensus view of the expert group regarding the relevance of each connection.

To ensure robustness, we also considered alternative thresholds of 65% and 75% and re-estimated the network structure under these conditions. The results indicated that while there were slight changes in network density and the relative centrality of certain stakeholders, the core stakeholders and the overall network structure remained stable across these thresholds.

This binary coding approach was adopted for clarity and simplicity in the network representation, as it helped to focus on the presence or absence of influence relationships that were widely acknowledged by the experts. However, we acknowledge that this approach may result in a loss of information about the intensity of relationships. Future studies could consider employing a weighted network approach to capture the strength of these ties in more detail.

Based on these criteria, the stakeholder relationship matrix was constructed and imported into UCINET 6.0, a widely used software for social network analysis. The software generated the stakeholder network structure and provided key metrics such as network density, degree centrality, betweenness centrality, and closeness centrality. These measures offered both visual and quantitative insights into how stakeholders interact and influence one another within the HMS, supporting a more rigorous interpretation of stakeholder dynamics.

## 4 Results

### 4.1 Demographic of respondents

A total of 631 experts participated in the questionnaire survey, with a mean age of 38.3 years. The sample was intentionally designed to ensure diversity across both sectors and geographic regions within China to minimize selection bias. The geographic distribution was as follows: 35% from eastern China, 33% from central China, and 32% from western China. Among the respondents, 35.02% were from government departments, 45.01% from healthcare organizations, 9.51% from industry associations, and 10.46% from other sectors (academia, patient advocacy, etc.). Of the 700 electronic questionnaires distributed, 631 were returned, yielding a response rate of 90.14%.

### 4.2 Stakeholder identification

A total of 14 stakeholders were identified as relevant to the HMS: the Government (59.3%), Finance Bureau (63.0%), Development and Reform Commission (50.6%), Bureau of Human Resources and Social Security (59.3%), Establishment Department (57.8%), Health Committee (76.0%), Medical Products Administration (67.0%), Healthcare Security Administration (71.7%), tertiary hospitals (61.0%), secondary hospitals (63.6%), community healthcare centers (69.7%), township health centers (76.3%), village clinics (71.9%), and patients or patient-related personnel (71.9%). These stakeholders formed the basis for subsequent analysis of influence relationships and network structure within the HMS.

### 4.3 Overall network-level characteristics

(1) *Network density and cohesion*. The analysis reveals a network density of 59.3%, indicating moderate connectivity. This suggests that while meaningful relationships exist among the 14 stakeholders, the network is not yet fully integrated, leaving room for enhanced information and resource exchange. The overall network structure shows a core-periphery configuration, where a dense cluster of administrative and financial bodies dominates the system' s interactions. (2) *Network centralization*. The centralization indices provide insight into the hierarchy and structural dependence of the network (Table 3). The Betweenness Centralization index is 8.91%, reflecting a decentralized brokerage structure where the “bridge” roles are not concentrated in a single entity, though specific actors dominate. The Degree Centralization for outdegree (43.787%) is significantly higher than that for indegree (27.219%), suggesting that the initiation of influence and directives is more concentrated in a few “hub” institutions than the reception of information.

**Table 3:** Network-level centralization results.

**Metric**	**Value**
Network density	59.34%
Network centralization (out-degree)	43.79%
Network centralization (in-degree)	27.22%
Network centralization (betweenness)	8.91%

### 4.4 Individual node-level characteristics

(1) *Degree and betweenness centrality*. The Healthcare Security Administration and Finance Bureau exhibit the highest normalized out-degree (100.00%), identifying them as the most active initiators of resource and policy flow. Conversely, Tertiary Hospitals exhibit the highest normalized in-degree (84.62%), indicating their role as major recipients of directives and referrals. Crucially, the Health Committee records the highest Normalized Betweenness Centrality (10.79%), confirming its unique status as the system's “broker”. It bridges the gap between the administrative core and service providers, managing the flow of strategic information. In contrast, grassroots organizations including Community Health Centers, Township Health Centers, and Village Clinics all show zero betweenness centrality, indicating they are structurally excluded from intermediary coordination roles. (2) *Closeness centrality*. High out-closeness scores for the Government, Establishment Department, and Medical Products Administration (ranging from 92% to 100%) suggest that these actors can rapidly reach other stakeholders to disseminate regulations. Conversely, Community Health Centers and Village Clinics show the lowest closeness (7.14%), highlighting a significant “feedback gap” where information from the grassroots struggles to reach the central decision-makers efficiently (Table 4).

**Table 4:** Standardized node centrality metrics for HMS stakeholders.

**Stakeholders**	**Normalized degree (Out)**	**Normalized degree (In)**	**Normalized betweenness**	**Normalized closeness (Out)**
Government	92.31%	46.15%	4.30%	92.86%
Finance bureau	100.00%	61.54%	6.28%	56.52%
Development and reform commission	100.00%	30.77%	0.24%	100.00%
Bureau of human resources and social security	76.92%	38.46%	0.08%	81.25%
Establishment department	92.31%	30.77%	2.89%	92.86%
Health committee	69.23%	61.54%	10.79%	76.47%
Medical products administration	69.23%	76.92%	2.29%	100.00%
Healthcare security administration	100.00%	61.54%	4.41%	68.42%
Tertiary hospitals	46.15%	84.62%	0.56%	100.00%
Secondary hospitals	23.08%	69.23%	0.48%	40.63%
Community healthcare centers	0.00%	69.23%	0.00%	7.14%
Township health centers	7.69%	76.92%	0.00%	76.47%
Village Clinics	0.00%	61.54%	0.00%	7.14%
Patients and patient-related personnel	53.85%	61.54%	3.77%	65.00%

### 4.5 Visualization analysis

**Figure 2:**
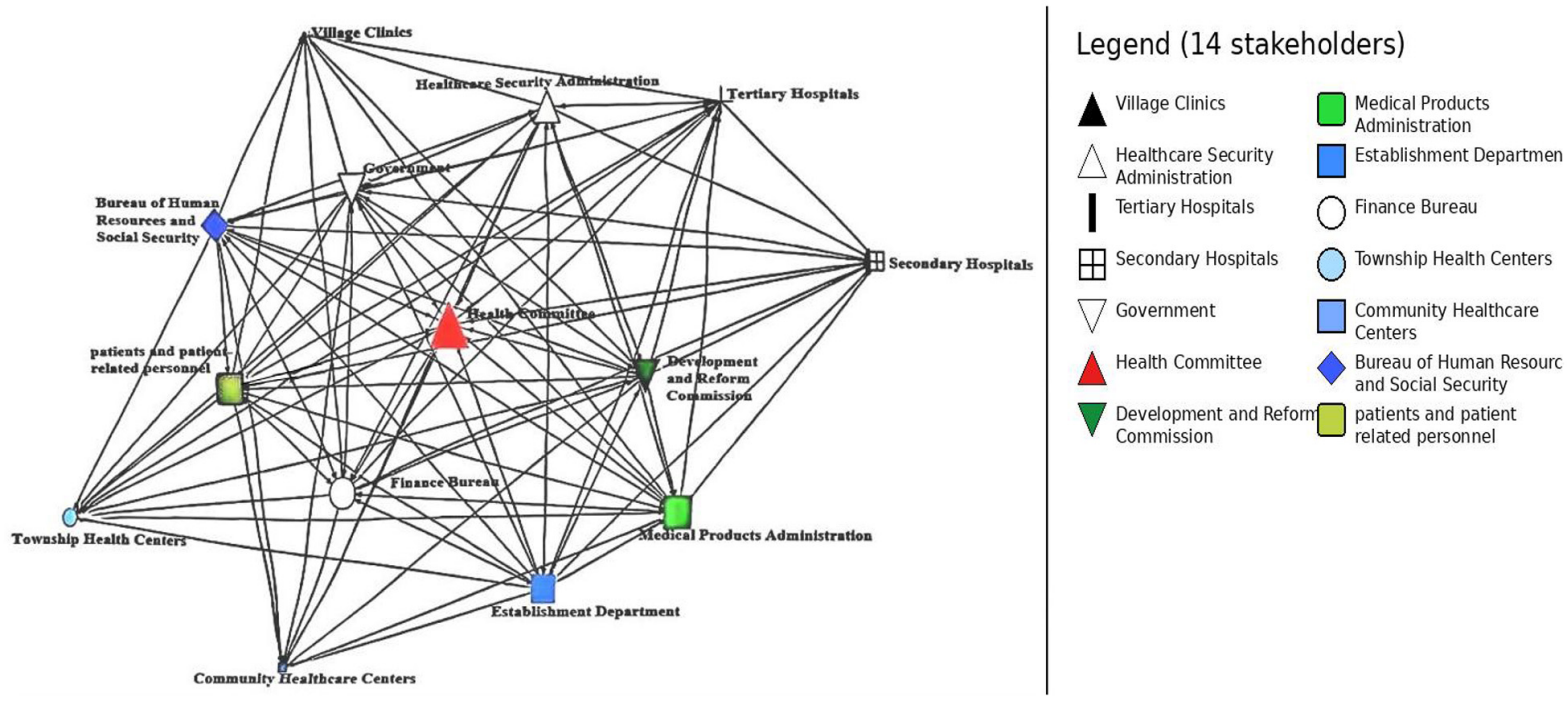
Relationship network structure diagram of stakeholders in HMS.

Figure 2 illustrates the stakeholder relationship network within the HMS, where node sizes are proportional to their normalized betweenness centrality and colors/shapes represent specific institutional roles. This visualization transitions from a schematic diagram to a substantive analytical tool, revealing the following structural insights: (1) *The administrative and brokerage core*. As shown in Figure 2, the Health Committee (marked in red) occupies the most prominent central position with the largest node size. This visually confirms its role as the primary “broker” and structural anchor of the HMS. Its high betweenness centrality indicates that it fills critical structural holes, bridging the gap between high-level policy-making bodies and frontline service providers. Similarly, the Government, Finance Bureau, and Healthcare Security Administration are positioned in the immediate “core” of the network. Their proximity and thick connecting ties reflect a “strong-tie” power cluster that dominates resource mobilization and policy formulation. (2) *Regulatory and human resource drivers*. The Development and Reform Commission, Medical Products Administration, and Establishment Department are strategically clustered near the administrative core. While the NDRC shows a high capacity for initiating connections (high out-degree), its relatively smaller node size compared to the Health Committee suggests that while it is a powerful policy driver, it serves less as a daily intermediary in the network' s communication flow. (3) *Peripheral service delivery and coordination bottlenecks*. The healthcare service delivery tier—comprising Tertiary Hospitals, Secondary Hospitals, Community Healthcare Centers, Township Health Centers, and Village Clinics—is positioned in the network's periphery. Notably, the grassroots providers are represented by the smallest nodes and exhibit sparse connectivity to the central brokers. This visual “peripheralization” highlights a significant coordination bottleneck: while policy directives flow downward from the core, the structural distance and lack of intermediary power (zero betweenness) at the grassroots level suggest that bottom-up feedback loops are severely constrained. (4) *Beneficiary integration*. Patients and Patient-related Personnel are positioned in the lower-left periphery. Although they possess moderate degree centrality, their distance from the administrative “brokerage” core (the Health Committee) suggests that patient experiences and feedback may not be efficiently translated into structural policy changes without passing through multiple bureaucratic layers.

## 5 Discussion

The analysis of the HMS stakeholder relationship network reveals several key insights into the roles and interactions of various stakeholders within the system. By employing network analysis metrics such as degree centrality, betweenness centrality, and closeness centrality, we can discern the network structure and the flow of influence and information across stakeholders. These metrics collectively provide a comprehensive view of the hierarchical nature of the network and the concentration of power within the system. In the following, we use these empirical findings as the basis for a collaborative “driving force-structure-process” lens to interpret how collaboration is (and is not yet) organized within the HMS.

### 5.1 Driving force: centralized power and passive periphery

Degree centrality analysis highlights that core stakeholders, including the Healthcare Security Administration, Finance Bureau, and Development and Reform Commission (all with 100% normalized out-degree) serve as the primary “engines” of the network. These stakeholders exhibit high levels of engagement in initiating policy and resource flows, representing the dominant “driving force” in our framework. This finding aligns with Etemadi et al. (24), who identified similar centralization in Iran's health system, where core governance entities dominate financial and informational flows. In contrast, the “feedback driving force” remains weak. Peripheral stakeholders, such as Village Clinics, Township Health Centers, and Community Health Centers, are typically passive information recipients (high in-degree but near-zero out-degree). This mirrors observations by Behzadifar et al. (25), where service providers remain marginalized and dependent on central bodies, suggesting that the impetus for HMS implementation is currently a top-down mandate rather than a collaborative, multi-directional effort.

### 5.2 Process: brokerage positions and structural holes

A critical finding regarding the “process” dimension is the identification of stakeholders occupying “structural holes”—the gaps between non-redundant contacts. The Health Committee, possessing the highest normalized betweenness centrality (10.79%), acts as the primary broker (intermediary) in the system. By bridging otherwise disconnected clusters—such as linking the Finance Bureau with primary healthcare providers—the Health Committee controls the flow of strategic information and fills structural holes that would otherwise leave the system fragmented. However, the analysis also reveals significant “coordination bottlenecks”. While the administrative core is tightly knit, the primary-level providers (Township Health Centers, Community Health Centers, and Village Clinics) all register zero betweenness centrality. This indicates a total lack of brokerage power at the grassroots level; they are isolated from other departmental clusters and from each other. These structural holes between the administrative core and the rural/community periphery suggest that resources may be trapped within the core, failing to reach the “last mile” of the HMS effectively. This result is consistent with Romiti et al. (26), who found that central actors, while essential for bridging, often inadvertently exacerbate peripheral isolation. Identifying these gaps reveals strategic leverage points where digital interventions or policy mandates could be used to “fill” these holes, fostering a more resilient and integrated process (27).

### 5.3 Structure: efficiency and feedback gaps

The closeness centrality analysis confirms the hierarchical “structure” of the network. The Health Committee and Medical Products Administration possess high out-closeness values, enabling them to disseminate directives with high efficiency. This aligns with Tenbensel et al. (28), who noted that high closeness in central actors facilitates rapid top-down flow but often limits bottom-up contributions. The isolated roles of Community Health Centers and Village Clinics (lowest out-closeness at 7.14%) reflect a structural “feedback gap”. This isolation is consistent with systematic reviews highlighting how peripheral stakeholders often face insurmountable challenges in influencing system-level decisions (17). Together with the pronounced core–periphery configuration, these results describe a network where information processes are heavily channeled through a narrow core, leaving peripheral actors functionally distant from the policy-making center.

### 5.4 Synthesis and implications for the Chinese context

The HMS network is dominated by a centralized core of governance and regulatory bodies, while service delivery organizations remain reactive endpoints. This conclusion is in line with Martinsen et al. (29), who analyzed EU health governance networks and emphasized the importance of integrating peripheral national providers to reduce imbalances and enhance equity, as well as Hunter et al. (30), who used stakeholder network analysis in non-communicable disease plans to highlight how centralized control can be mitigated through inclusive policy-making. In the Chinese context, these findings provide a system-level explanation for persistent obstacles in tiered diagnosis and treatment, such as the difficulty in strengthening the “gatekeeping” role of primary care (31). Despite policies promoting county-medical communities, our data shows that Township Health Centers and Village Clinics still lack the brokerage power (zero betweenness) necessary to coordinate care across levels (32). The “periphery” status of these institutions explains why policy effects—such as those seen in family doctor services—often weaken over time (33); they lack the structural “connective tissue” to sustain influence within the broader bureaucratic network.

### 5.5 Interpretive lens: beyond node equivalence

Interpreted through the driving force-structure-process framework, these metrics are not abstract values but indicators of practical implementation. The “driving force” is the concentrated authority of the Finance Bureau and Development and Reform Commission; the “structure” is the core-periphery divide; and the “process” is the Health Committee' s brokerage role in filling structural holes. Finally, while stakeholders like Patients are represented as equivalent nodes, they should not be viewed as identical in power to state agencies. Their position in the lower-left periphery, distant from the brokerage core, visualizes how expert consensus perceives the “indirect” influence of patient feedback. Including them in the network allows us to see the structural distance between public experience and administrative “brokers”, highlighting the need for more direct pathways to integrate patient and grassroots feedback into the core coordination process.

## 6 Suggestions

Based on the findings from the HMS stakeholder relationship network analysis, which highlight a centralized structure with limited integration of peripheral actors, we propose targeted suggestions to foster a more equitable and collaborative healthcare system. These suggestions are framed within the collaborative driving force-structure-process framework, a widely adopted framework in network governance literature for enhancing stakeholder interactions in complex systems like healthcare. This model emphasizes the interplay between motivational drivers, organizational structures, and operational processes to promote effective collaboration. By addressing these elements, the HMS can transition from a hierarchical model to one that empowers peripheral stakeholders, improves information flow, and enhances overall system resilience.

### 6.1 Collaborative driving force

To cultivate stronger collaborative driving forces, policymakers should prioritize incentives that motivate both central and peripheral stakeholders to engage actively in the network. For instance, introducing shared performance metrics—such as joint accountability for healthcare outcomes at the community level—could align interests and encourage core entities like the Healthcare Security Administration and Finance Bureau to involve peripheral actors (e.g., village clinics and township health centers) in resource allocation decisions. Additionally, external drivers, such as regulatory mandates for inclusive policy dialogues or funding tied to collaborative benchmarks, could stimulate participation from isolated stakeholders. These measures would address the current imbalance where peripheral actors are passive recipients, fostering intrinsic motivations like mutual trust and shared goals. This strategy is supported by research on healthcare network governance, where motivational drivers like policy incentives and resource dependencies have proven effective in boosting stakeholder involvement. For instance, Etemadi et al. (24) illustrated in Iran's health system that leveraging network governance theories with financial support mechanisms as drivers can foster equity by incentivizing peripheral participation.

### 6.2 Collaborative structure

Reconfiguring the collaborative structure entails decentralizing the HMS network to diminish dependence on central brokers and better incorporate peripheral stakeholders. A primary recommendation is to adopt hybrid governance approaches, such as co-led committees where peripheral entities jointly oversee sub-groups focused on service delivery and policy input. This could involve creating regional nodes that directly link township health centers and community healthcare centers to core institutions like the Health Committee, thereby reducing hierarchy and elevating the betweenness centrality of peripheral actors. Additionally, deploying digital tools for network visualization and instant connectivity could address structural disparities, granting greater visibility and agency to marginalized stakeholders. These structural changes are corroborated by studies examining healthcare networks. Romiti et al. (26) observed in Italian cancer networks that hybrid structures enhance coordination by empowering peripheral providers, while Larrain et al. (27) demonstrated through social network analysis that integrated structures improve cooperation and alleviate inefficiencies stemming from centralization.

### 6.3 Collaborative process

Improving collaborative processes involves standardizing protocols for communication, decision-making, and dispute resolution in the HMS. Key suggestions include organizing periodic multi-stakeholder workshops for ongoing feedback cycles, enabling peripheral actors to influence policy development via formalized data exchange and dialogue. Capacity-building initiatives, such as training in negotiation and information sharing, could equip village clinics and community centers for more proactive roles. Furthermore, introducing tools like consensus-oriented sessions would accelerate information flow, countering the low closeness centrality observed in peripheral nodes. Such process enhancements are drawn from evidence in public health networks. Tenbensel et al. (28) showed in New Zealand's health policy implementation that inclusive processes build policy capacity and alleviate isolation, whereas Martinsen et al. (29) noted in EU health governance that transparent decision-making integrates peripheral stakeholders to yield superior outcomes.

### 6.4 Feasibility and barriers to implementation

While these suggestions are designed to foster a more collaborative and equitable system, the feasibility of implementing these changes faces several challenges. The entrenched hierarchical nature of the current system poses a significant barrier, as core stakeholders like the Health Committee and Government hold substantial power and may be resistant to decentralizing authority. Additionally, the capacity of peripheral stakeholders, particularly primary care institutions, is often limited by financial constraints and insufficient resources, which may hinder their active participation in collaborative processes. Moreover, cultural and organizational differences between stakeholders could affect trust-building and the effectiveness of new governance structures.

Overcoming these barriers will require targeted efforts to realign interests, build trust, and provide resources and training to ensure that peripheral stakeholders are empowered to take on more active roles in governance. Additionally, creating a shared vision for healthcare reform across stakeholders and building institutional capacity at the grassroots level will be key to overcoming resistance and ensuring the success of these collaborative reforms.

## 7 Conclusions

This study elucidates the intricate stakeholder relationship network within the HMS through social network analysis, revealing a highly centralized structure dominated by core entities such as the Healthcare Security Administration, Finance Bureau, and Health Committee. Metrics including degree centrality, betweenness centrality, and closeness centrality underscore the pivotal roles of these central actors in information dissemination, policy coordination, and resource allocation, while peripheral stakeholders, such as village clinics and community healthcare centers, remain isolated and passive, limiting overall system equity and collaboration. These findings contribute to the growing body of literature on healthcare network governance by highlighting the implications of structural imbalances for service delivery and policy efficacy, particularly in hierarchical systems akin to those examined in prior works.

The proposed suggestions, framed within the collaborative driving force-structure-process model, offer practical pathways to decentralize the network, incentivize peripheral engagement, and streamline processes for enhanced integration. By fostering a more equitable HMS, this approach can improve healthcare accessibility, responsiveness, and resilience, ultimately benefiting underserved communities. However, limitations such as reliance on self-reported data and the static nature of the analysis warrant caution in generalizing results. Future research should employ longitudinal designs and incorporate dynamic modeling to evaluate the long-term impacts of these interventions on network evolution and health outcomes.

## 8 Limitations

Several limitations should be acknowledged to ensure a balanced interpretation of our findings.

### 8.1 Methodological and data constraints

Although we clarified the search scope and eligibility criteria for the literature review, the final set of 13 articles remains relatively small and may be influenced by database selection and screening rules. The threshold of 50% expert endorsement for stakeholder categories, while pragmatic, may introduce selection bias. Furthermore, the study relies on expert judgment for defining both the node set and influence ties. While we used a two-round procedure, independent validation through triangulation with policy documents, administrative records, or field interviews would strengthen the findings. Additionally, since the relationships are based on self-reported perceptions, they may be subject to social desirability bias, potentially overstating coordination or under-reporting conflicts.

### 8.2 Analytic and static nature

The use of a binary adjacency matrix based on consensus thresholding inevitably discards information regarding relationship intensity. While sensitivity checks suggest a stable core structure, these choices affect network density. Moreover, as UCINET provides descriptive point estimates without standard errors for a single network, minor differences in centrality scores should not be over-interpreted. Crucially, this analysis is cross-sectional and static, providing a snapshot of the HMS that may not capture the dynamic evolution of relationships driven by ongoing policy adjustments and organizational restructuring.

### 8.3 Systemic complexity and regional variations

This study constitutes a comprehensive observational analysis at the national level, which may mask the systemic complexity and profound regional variations inherent in the Chinese healthcare landscape. The implementation of the HMS varies significantly across provinces and municipalities—for instance, between the “Sanming model” (characterized by deep integrated reform) and the “Shanghai model” (focused on family doctor contract services). Furthermore, marked disparities across administrative levels mean that a national aggregate model might overlook nuanced local dynamics and specific resource-sharing constraints unique to certain geographic or economic contexts.

### 8.4 The impact of digital intervention

Our static SNA model may not fully capture the influence of rapid digital health interventions on stakeholder interconnections. The introduction of “Internet + Healthcare” initiatives and regional health information platforms has created “virtual” ties and new communication pathways that sometimes bypass traditional bureaucratic hierarchies. These digital tools may reshape the brokerage roles and structural holes identified in this study. Future research should adopt dynamic or longitudinal designs to track how digital transformation and regional policy experiments reshape stakeholder networks over time, moving toward a more granular and multi-level understanding of HMS governance.

## Data Availability

The original contributions presented in the study are included in the article/Supplementary material, further inquiries can be directed to the corresponding author.
