# Supplementary material for: Social network analysis of stakeholders in China's hierarchical medical system: toward a collaborative governance framework for enhanced integration
Source: Front Public Health. 2026 Mar 11;14:1720264. doi: 10.3389/fpubh.2026.1720264 (PMC13013354; doi:10.3389/fpubh.2026.1720264)
Supplement: Supplementary file 1 [file Table_1.doc]

Version：001

Version Date：2023-4-28

**Stakeholders’ Collaboration in Hierarchical Medical System Survey Questionnaire**

**Part I. Basic information on participants**

1. Name of your workplace：

2. Type of your organization：

A. government medical administration B. health administration C. health insurance D. health care organization F. others

3. Number of years you have been in the workforce：

A. less than 2 years B.2-5 years C.5-10 years D. more than 10 years

1. Your professional title：
2. Senior B. Deputy Senior C. Intermediate D. Junior

**Part II Stakeholder Identification for the Collaborative of Hierarchical Medical System**

A stakeholder is any person or group of persons who can affect the achievement of an organization's objectives and who can be affected by the process of achieving those objectives, and should therefore be defined in terms of "affecting or being affected by the organization's activities" and "having a relevant interest".

The county hierarchical medical system stakeholders refer to the individuals, groups and institutions that can, to varying degrees, influence the realization of the goals of patients' first visit to primary community health centers and two-way referrals in the process of the construction of the medical community, or are influenced by the activities and policies of the operating hospitals.

The purpose of this part of the research is to fully identify the stakeholders of the hierarchical medical system. Please select whether the individuals, groups and organizations listed in the table are the stakeholders of hierarchical medical system, and tick “√” if they are, or tick “×” if they are not. If you have additional candidate stakeholders, please add them in the space provided.

| Stakeholders | Whether or not they are stakeholders |
| --- | --- |
| Health Committee |  |
| Finance Bureau |  |
| Healthcare Security Administration |  |
| Government |  |
| Patients and Patient-related Personnel |  |
| Establishment Department |  |
| Medical Products Administration |  |
| Tertiary Hospitals |  |
| Secondary Hospitals |  |
| Development and Reform Commission |  |
| Bureau of Human Resources and Social Security |  |
| Township Health Centers |  |
| Village Clinics |  |
| Community Healthcare Centers |  |
| Stakeholders |  |
| Health Committee |  |
| Other stakeholders added |  |

**Part Ⅲ Identify the main stakeholders in Hierarchical Medical System**

Instructions for filling in the form:The following table shows the stakeholders involved in the process of building the county hierarchical diagnosis and treatment, and the relationship between these stakeholders may have an influence on each other. Please judge whether there is any influence between these stakeholders according to the actual situation in the process of county medical community construction. Use "√" and "×" to indicate the influencing relationship between the stakeholders in the process of building the county medical community, if there is an influencing relationship between them, then it is "√"; on the contrary, it is √" if there is a relationship of influence between them; otherwise, it will be "×". Please indicate your judgment in the corresponding box in the table below.

| Stakeholders | Health Committee | Finance Bureau | Healthcare Security Administration | Government | Patients and Patient-related Personnel | Establishment Department | Medical Products Administration | Tertiary Hospitals | Secondary Hospitals | Development and Reform Commission | Bureau of Human Resources and Social Security | Township Health Centers | Village Clinics | Community Healthcare Centers |
| --- | --- | --- | --- | --- | --- | --- | --- | --- | --- | --- | --- | --- | --- | --- |
| Health Committee |  |  |  |  |  |  |  |  |  |  |  |  |  |  |
| Finance Bureau |  |  |  |  |  |  |  |  |  |  |  |  |  |  |
| Healthcare Security Administration |  |  |  |  |  |  |  |  |  |  |  |  |  |  |
| Government |  |  |  |  |  |  |  |  |  |  |  |  |  |  |
| Patients and Patient-related Personnel |  |  |  |  |  |  |  |  |  |  |  |  |  |  |
| Establishment Department |  |  |  |  |  |  |  |  |  |  |  |  |  |  |
| Medical Products Administration |  |  |  |  |  |  |  |  |  |  |  |  |  |  |
| Tertiary Hospitals |  |  |  |  |  |  |  |  |  |  |  |  |  |  |
| Secondary Hospitals |  |  |  |  |  |  |  |  |  |  |  |  |  |  |
| Development and Reform Commission |  |  |  |  |  |  |  |  |  |  |  |  |  |  |
| Bureau of Human Resources and Social Security |  |  |  |  |  |  |  |  |  |  |  |  |  |  |
| Township Health Centers |  |  |  |  |  |  |  |  |  |  |  |  |  |  |
| Village Clinics |  |  |  |  |  |  |  |  |  |  |  |  |  |  |
| Community Healthcare Centers |  |  |  |  |  |  |  |  |  |  |  |  |  |  |
| Other stakeholders added |  |  |  |  |  |  |  |  |  |  |  |  |  |  |
